# Effects of cold exposure on behavioral and electrophysiological parameters related with hippocampal function in rats

**DOI:** 10.3389/fncel.2014.00253

**Published:** 2014-09-01

**Authors:** Hajar Elmarzouki, Youssef Aboussaleh, Soner Bitiktas, Cem Suer, A. Seda Artis, Nazan Dolu, Ahmed Ahami

**Affiliations:** ^1^Laboratory of Nutrition and Health, Department of Biology, Faculty of Science, Ibn Tofail UniversityKenitra, Morocco; ^2^Department of Physiology, Erciyes University School of MedicineKayseri, Turkey; ^3^Department of Physiology, Medeniyet University School of Medicineİstanbul, Turkey

**Keywords:** stress, hippocampus, long-term potentiation (LTP), Morris water maze test (MWM), rat

## Abstract

**Aim**: Behavioral and mental changes may occur in people exposed to cold stress by decreasing their work efficiency and their mental capacity while increasing the number of accidents on the job site. The goal of this study was to explore the effect of cold stress in spatial learning performance excitability and LTP.

**Materials and Methods**: Three to four month old rats were randomly divided into four groups to form a control group and a cold stress group for each sex. The groups of cold stressed animals were placed in a cold room ambient temperature of 4°C for 2 h day. Adrenal glands and body weight (g) were recorded in control and stressed rats during the cold exposure. Spatial learning (acquisition phase) and memory (probe trial) were tested in the Morris water maze (MWM) immediately after daily exposure. Latency to locate the hidden platform, distance moved (DM), mean distance to platform, swim speed (SS) and time spent in the platform quadrant were compared between genders and treatments. Field potential recordings were made, under urethane anesthesia, from the dentate gyrus (DG) granule-cell layer, with stimulation of the medial perforant pathway 2 h after the probe trial. This study examined spatial memory as measured by MWM performance and hippocampal long-term potentiation (LTP) in the DG after exposure to cold in a repeated stress condition for 2 h/day for 5 days.

**Results**: The cold-exposed female rats needed less time to find the hidden platform on day 1 (43.0 ± 13.9 s vs. 63.2 ± 13.2 s), day 2 (18.2 ± 8.4 s vs. 40.9 ± 12.2 s) and on day 4 (8.0 ± 2.1 s vs. 17.2 ± 7.0 s) while cold-exposed male rats showed a decreased escape latency (EL) on day 1 only (37.3 ± 12.5 s vs. 75.4 ± 13.1 s). Cold-exposed male rats spent less time in the target quadrant (30.08 ± 6.11%) than the control male rats (37.33 ± 8.89%). Two hour cold exposure decreased population spike (PS) potentiation during both induction (218.3 ± 21.6 vs. 304.5 ± 18.8%) and maintenance intervals (193.9 ± 24.5 vs. 276.6 ± 25.4%) in male rats. Meanwhile cold exposure did not affect the body weight (C: 221 ± 2.5 vs. S: 222 ± 1.7) but it impacts the adrenal gland relative weight (S: 27.1 ± 1.8 mg vs. C: 26.2 ± 1.4 mg).

**Conclusion**: Overall, the results show that repeated cold exposure can selectively improve spatial learning in adult female rats, but impaired retention memory for platform location in male rats. It is possible that impaired LTP underlies some of the impaired retention memory caused by cold exposure in the male rats.

## Introduction

People who live mainly in cold countries and outdoor workers are exposed to hazardous cold stress. Indeed cold may decrease mental capacity attentiveness, work efficiency and increase the number of accidents on the jobsite. Other behavioral alterations like a reduced mental alertness, craziness and confused behavior were also reported.

From a psychological point of view, cold causes unpleasant thermal sensations or even cold-induced pain, which is experienced as stress. Exposure to stress is associated with an alteration in learning and memory formation in experimental animals and human subjects (Zheng et al., 2008). Although the performance of stressed animals in a learning task critically depends on the type of stressor, cold as a stressor has been relatively less used. Apart from metabolic stressors such as hypoglycemia, glucopenia, and emotional stressors, which cause adrenaline release by activating the sympatho-adrenomedullary system, cold exposure (as well as pain) causes noradrenaline release from the sympathetic terminals (Kvetnanský et al., 1997; Kvetnansky et al., 2009). Afferent nerves from cold receptors, located in the skin, terminate in the hypothalamus and then multisynaptic pathways reach limbic areas, especially the hippocampus, leading to noradrenaline release (Rintamaki, 2005; Kvetnansky et al., 2009). Considerable evidence supports a close interaction between stress and the noradrenergic system in the hippocampus (Nisenbaum et al., 1991; Britton et al., 1992; Sandi et al., 2005).

The existence of sex differences in the standard rat and mouse models of learning and memory is a controversial topic in the literature. Although a few studies used females to examine the influence of stress on hippocampal functions focusing on gender difference in learning and memory, findings indicate male advantage for rats in radial maze and water maze protocols (Jonasson, 2005). As a general observation, the larger adrenaline responses to stress found in females than in males can be explained by the different hippocampal cognitive ability in both sexes in response to stress. However a similar difference in noradrenaline is rarely observed.

To the best of our knowledge, there is no study examining the effect of intermittent cold stress from behavioral and/or electrophysiological aspects. In the present study we measured the spatial memory performance of adult male and female rats using the Morris water maze (MWM). We also recorded Long-Term Potentiation (LTP) from the dentate gyrus (DG), a model of synaptic plasticity that is thought to underlie learning and memory processes (Bliss and Collingridge, 1993), as an electrophysiological correlate of behavior. Both tests used in this study require hippocampal integrity (Morris et al., 1982; Sutherland et al., 1982) and has been extensively employed in studying the relationship between hippocampal function and spatial learning and memory in rodents (Rosenzweig and Barnes, 2003).

The goal of our study was to explore the effect of cold stress in spatial learning performance excitability and LTP, and the results obtained may explain the behavioral and mental changes that may occur in people exposed to cold.

## Materials and methods

### Animal care

The experiments were conducted in compliance with the guiding principles for the care and use of laboratory animals approved by Erciyes University. In this study, thirty-six male and female rats (3–4 months old) weighing 200–250 g, were randomly divided into four groups to form a control group and a cold stress group for each sex. The groups of cold stressed animals were placed in a cold room (ambient temperature of (4°C) for 2 h/day between 8:00 a.m. and 10:00 a.m. to avoid corticosterone circadian rhythm. The acclimation of control animals was according to standard animal laboratory conditions (12 h/12 h light/dark cycle, temperature 22°C).

### Body weight and adrenal gland weight

In order to determine the effect of cold stress procedures, the body weight and the adrenal gland weight were measured in control and stressed rats.

### Rectal temperature

We examined changes in rectal temperature due to stress. Rectal temperature was digitized for 1-min with a probe (Biopac) immediately after cold exposure in the stress groups. The mean temperature of 1 min for each rat was averaged.

### Behavioral testing

#### Morris water maze testing

The evaluation of performance in the MWM was used to measure spatial memory. The experiments were realized in a circular, galvanized steel maze (2 m in diameter and 75 cm in depth), which was filled with water at a depth of 50 cm and kept at 22°C. A blue non-toxic dye was added to the water to make it opaque. The maze was placed in a large quiet test room, surrounded by many visual cues external to the maze (some A3 sized posters), which were visible from within the pool and could be used by the rats for spatial orientation. There was no change in the locations of the cues during the period of testing. Four equal quadrants were divided in the pool. In one of the quadrants, a platform (10 cm in diameter) was placed centrally and fixed in a position which was kept constant during the acquisition trials.

An automated video-tracking system (Noldus) recorded the position of the rat in the tank. A camera (TOTA-450III, Japan) was mounted 1.5 m above the surface of the water and was connected to a computer. Light was provided by four 40-W fluorescent lamps, mounted in a square pattern, 1.4 m above the surface of the water (Liu et al., 2006). Each trial was started manually and ended automatically when the rat escaped on the platform. All the trials were completed between 10.00 to 12.00 h (after cold exposure) and the experimenter always stood at the same position to avoid technical bias.

##### Reference memory

These trials have trained the rats to find a hidden platform and have helped them to remind their constant position throughout the training days; within 120 s. Rats underwent four trials per day for four consecutive days in the reference memory version of the MWM test.

The launch of each rat in the water was slow, and at a random quadrant except the target one that contained the platform. After reaching the platform, the rat was allowed to stay on it for 15 s and was then put back into its cage. If they could not escape to the platform within 120 s by themselves, the rat was placed on the platform by hand for 15 s. The rats were kept in a dry home cage for 60 s during the inter-trial intervals.

##### Memory consolidation

A probe test was carried out the day after the acquisition phase. The platform was removed and the rats were allowed to swim freely in the pool for 60 s. The degree of memory consolidation acquired after learning was indicated by the time in seconds spent in the target quadrant, which had contained the hidden platform.

#### Electrophysiology

##### Stimulation and recording

Electrophysiological responses are recorded at room temperature (approximatley 22°C). The Urethane (1.5 g/kg, ip) was used to anesthetize the rats and they were placed in a stereotaxic frame (Kopf Instruments, Tujunga, CA, USA). The stimulation of the medial perforant path (from bregma, in mm: AP: −7.0, ML: 4.2, DV: 2–2.5 below dura) of the right hemisphere was performed by a bipolar tungsten electrode (stainless steel, Teflon coated, 127 μm in diameter, insulated except at its tips). The output of a stimulus isolator (World Precision Instruments, USA) was connected to the Stimulating electrode. 3 M NaCl (tip resistance: 2–10 M) was used to fill a glass micropipette (Borosilicate, o.d.: 1.5 mm,10 cm length, World Precision Instruments). For recording field excitatory postsynaptic potentials (fEPSPs), the glass micropipette was inserted in the granule cell layer of the ipsilateral DG (in mm: AP: 3.5, ML: 2.15, DV: 2.5–3 mm below dura). The reference electrode was positioned under the neck skin (An Ag-AgCl disc electrode).

A head- stage was used to connect the recording and reference electrodes to an amplifier (VCC600 single channel epithelial voltage/current clamp system, Physiological Instruments). A Faraday cage was used to shield the entire system. To obtain a large positive fEPSP, the depth of recording electrode was adjusted, and a super imposed negative-going population spike (PS) was evoked with a 0.1 mm step. After recording a typical response, the final depth of the stimulating electrode was adjusted to maximize the PS amplitude in response to the perforant path stimulation.

To control stimulation and recording the “Scope” program (ADInstruments, Colorado Springs, CO, USA) was used. Monophasic 10 V and 0.175-ms pulses were generated by the A/D board (Powerlab/8SP, ADInstruments, Colorado Springs, CO, USA) of a computer and triggered to a stimulator which was connected to an isolator (Suer et al., 2009). Biological signals were amplified (1000×). A pre-amplifier allowed to amplify the biological signals (1000×) at a bandwidth of 0.1–10 kHz. Waveforms were digitized on-line at a rate of 40 kHz for 20 ms, displayed on a computer monitor, and stored using Scope for off-line analysis.

##### Input—Output (I/O) Curve

The stimulation consisting of 175 μs duration monophasic constant current pulses was delivered once every 20 s for 15 min after electrode placement, and it allowed to obtain an I/O curve. The stimulation current ranged from 0.1 to 1.5 mA. There was an average of three evoked responses for each current value. A sigmoidal curve described the relation between stimulus intensity and the fEPSP slope or PS amplitude; the half maximal stimulus intensity was determined from this curve. The stimulus intensity that produced 50% of the maximum response (i.e., test pulse) was used in subsequent experiments.

##### Long-term potentiation

Then, after a 15 min baseline recording of the fEPSPs, four sets of tetanic trains (high frequency stimulation: HFS, 100 Hz, 1 s), separated by 5 min intervals at 15, 20, 25 and 30 min, were administered to induce LTP. Following delivery of the last tetanic train, test stimuli were repeated every 30 s for up to 80 min (Artis et al., 2012).

##### Data analysis and statistics

The PS amplitude was measured from the first positive peak to the negative peak. The slope of the fEPSP was calculated as the amplitude change at 20–80 t% of the voltage difference between the start of the waveform and the fEPSP amplitude at the onset of PS. During the I/O experiments the maximum value of the fEPSP slope or PS amplitude was evaluated as 100 percent, the EPSP and PS was expressed as the percentage of this value. Analysis of two-way (Treatment: 22°C vs. 4°C × Gender: male vs. female) ANOVA with repeated measures (Stimulus intensity) was conducted on the EPSP slope and on the PS amplitude.

The mean value of the fEPSP slope or PS amplitude during the first 15-min period (30 sweeps) was evaluated as 100 percent and was defined as the baseline. Each EPSP and each PS was expressed as the percentage of the baseline value (Artis et al., 2012). The increase in EPSP slope and spike amplitude during HFS application (0–15 min) was considered as a measure of the induction of LTP. The remainder of the experiment (15–80 min) serves for the maintenance of LTP. A repeated-measures ANOVA with two between (Treatment × Gender) and one within (Interval: baseline, induction, and maintenance) subject factor was conducted on the EPSP slope and spike amplitude separately.

Escape latency (EL) was measured as the time in seconds between being placed in the water and finding the hidden platform. Distance moved (DM) was the distance of the swim path between the start location and the hidden platform (Liu et al., 2006). Mean distance from the platform (MDP) was also measured as an indicator of reference memory error, and swim speed (SS) as an indicator of the rat’s motor ability over 4 days. Results from the four trials for each training day were averaged for every rat and used for the statistical analysis. Analysis of two-way (Treatment × Gender) ANOVA with repeated measures (Day) was conducted on the EL, DM, MDP, and SS. Shortened EL, and decreased DM and MDP without changing SS across days was evaluated as outperformed learning performance.

A two factor ANOVA (Treatment × Gender) was conducted on the means of day 5 (probe trial) in terms of the time spent in the target quadrant and rectal temperature. The degree of memory consolidation which had taken place after learning was indicated by the time spent in the target quadrant.

The comparison between two independent groups was made using an least square difference (LSD) *Post hoc* test. The SPSS statistical software package version 10.0 (SPSS Inc., Chicago, Illinois, USA) performed all statistical analysis.

## Results

### Cold exposure decreased the rectal temperature in both male and female rats

Since we used a 2-h cold exposure to elicit the changes in spatial memory, we measured rectal temperature to determine whether such a stress protocol exerts a similar effect in both sexes. The rectal temperatures of the control male (*n* = 9) and female (*n* = 9) rats were 36.3 ± 0.31°C and 36.21 ± 0.29°C, respectively. After 2 h exposure to cold, the temperature of the male rats decreased to 32.73 ± 1.07°C, and that of the female dropped to 32.79 ± 0.97°C. An ANOVA showed significant Treatment Effect (*F*_(1,35)_ = 173.261; *p* < 0.001) with non significant main effect of gender (*F*_(1,35)_ = 0.004; *p* = 0.950) and non significant Interaction Gender × Treatment (*F*_(1,35)_ = 0.047; *p* = 0.787).

### Cold exposure effect on body weight and adrenal gland weight

**Figure 1:**
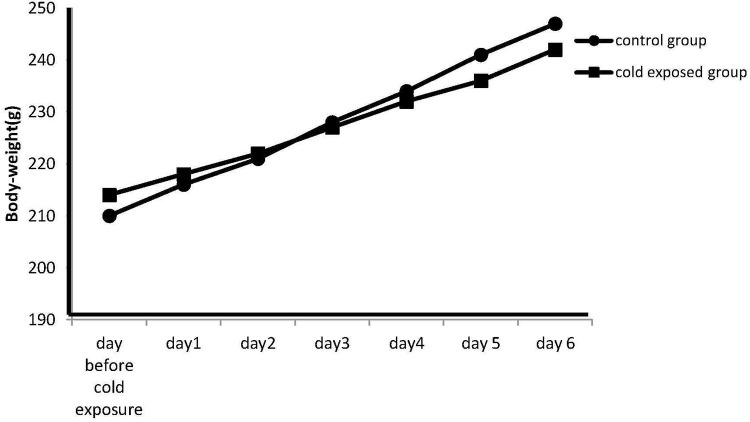
**Effect of cold stress on the body weight**. Data are represented as the mean S.E.M. of body-weight measured at 09:00 h daily before the exposure. Differences in body weight gain between controls and stressed rats are not statistically significant during any time period of the study (*P* < 0.07; *n* = 7 per group).However the *p* nearing 0.05 seem to suggest that increasing the number of observation the difference could be relevant.

The body weight on the day before cold stress exposure was 210 ± 2.3 g in the control group and 214 ± 2.9 g in the stressed group. Data are represented as the mean S.E.M. of body-weight measured at 09:00 h daily before the cold exposure. Differences between controls and stressed rats are not statistically significant at each experimental (*P* < 0.07; *n* = 7 per group; Figure 1; Day 1: 216 ± 2.1 vs. 218 ± 2.5; Day 2: 221 ± 2.5 vs. 222 ± 1.7; Day 3: 228 ± 1.6 vs. 227 ± 3.3; Day 4: 234 ± 2.1 vs. 232 ± 2.4; Day 5: 241 ± 2.7 vs. 236 ± 2.2; Day 6: 247 ± 2.9 vs. 242 ± 2.8). This *p* nearing 0.05 however seem to suggest that increasing the number of observations the difference could be relevant.

Daily exposure to 2 h cold for 5 days resulted in an insignifant increase in adrenal weight in the stressed animals (27.1 ± 1.8 mg vs. 26.2 ± 1.4 mg, *n* = 7 per group, *P* < 0.06). However the analysis with one-way ANOVA revealed a significant effect of stress on adrenal gland/body weight ratio at the time of autopsy (*P* < 0.009; 0.07 ± 0.01 mg organ/g of body weight S: 0.1 ± 0.02 mg organ/g of body weight).

### Behavioral measurements

#### Cold exposure improved acquisition performance in female rats, but, disrupted retrieval performance in male rats

**Figure 2:**
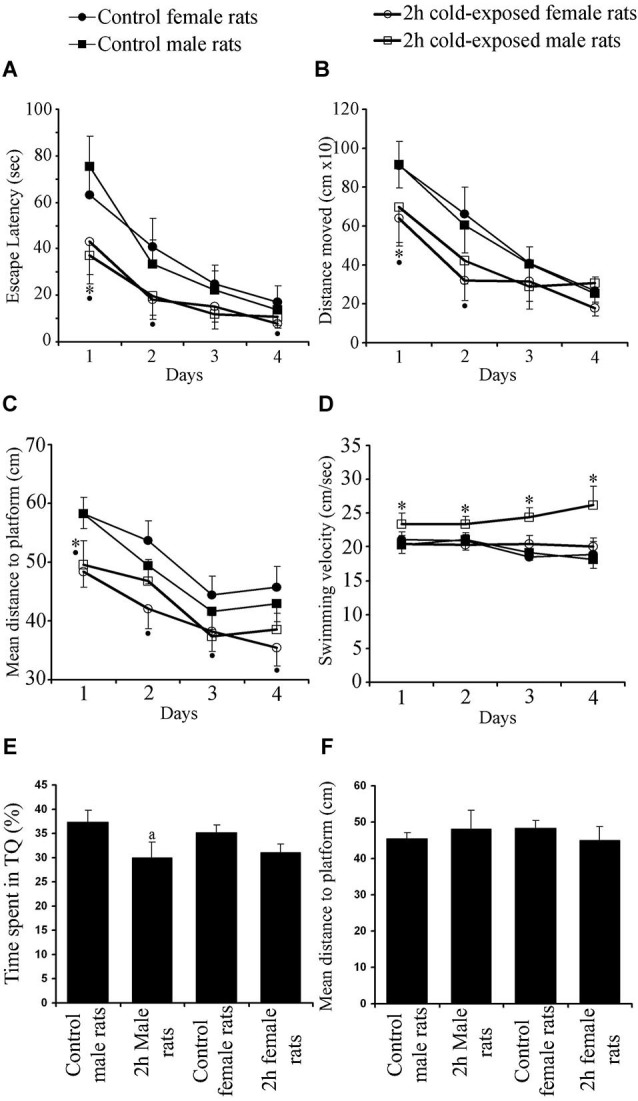
**Effects of cold exposure on acquisition performance in a task to find hidden platform in the water maze**. All rats improved their ability as shown by decreasing escape latency (A) and distance moved (B) over training days. Control rats swam further away from the platform than experimental rats on the 1st and 2nd days as shown by mean distance to the platform (C). Swimming speed (D) did not change as a function of day. Measures per testing day represent the average of four trials of all animals in each group. Each symbol represents the mean ± SE of 10–12 rats. * represents significant difference between cold exposed and control male rats; • represents significant difference between exposed and control female rats. Cold-exposed male rats spent less time in seconds in the target quadrant than the control male rats. Similar retrieval performance was observed between cold-exposed and control female rats (E). Mean distance to platform in the probe trial was not affected by Treatment or Gender (F).

In the MWM, which requires the integrity of the dorsal hippocampus, all groups successfully learned to find the hidden platform as shown by shortened ELs, DMs and MDPs (Day Effects: *F*s_(3,525)_ = 70.336, 78.515, 78.938; *P* < 0.001) over the four training days. The SS did not change within each group, showing similar motor ability (*F*_(3,525)_ = 1.729; *P* > 0.05) during the training period. A Repeated-Measures ANOVA showed significant Treatment Effect on EL, DM, MDP and SS (*F*s_(1,172)_ = 33.662, 19.531, 47.709 and 30.026; *P*s 0< 0.001). Significant Gender Effect was found on SS only (*F*_(1,172)_ = 17.340; *P* = 0.001) and significant Interaction Effects were found on MDP (*F*_(1,172)_ = 4.666; *P* = 0.032) and SS (*F*_(1,172)_ = 20.786; *P* = 0.001). LSD *post hoc* tests showed that 2-h cold exposure decreased EL (Figure 2A; 75.4 ± 13.1 vs. 37.3 ± 12.5; *P* = 0.001), DM (Figure 2B; 914.6 ± 118.0 vs. 697.5 ± 183.8; *P* = 0.034), and MDP (Figure 2C; 58.2 ± 2.5 vs. 49.6 ± 4.1; *P* = 0.001) on the first day of training in male rats.

During the remaining days of the training period no difference was observed between cold-exposed male and control male rats in any learning measurement, with observed trends on EL (*P*s = 0.058 and 0.052 on 2nd and 4th day, respectively) and MDP (*P*s = 0.077 and 0.054 on 3rd and 4th day, respectively). SS was faster in cold-exposed males than in control male rats on all training days (Figure 2D; Day 1: 20.3 ± 1.2 vs. 23.4 ± 1.6; Day 2: 21.1 ± 1.5 vs. 23.4 ± 1.2; Day 3: 19.2 ± 1.2 vs. 24.4 ± 1.3; Day 4: 18.2 ± 1.4 vs. 26.2 ± 2.8; *P*s 0 < 0.02). However cold-exposed female rats outperformed the task on the 1st day [as shown by EL (Figure 2A; 63.2 ± 13.2 vs. 43.0 ± 13.9; *P* = 0.035), DM (Figure 2B; 909.6 ± 126.2 vs. 639.8 ± 142.8; *P* = 0.009), and MDP (Figure 2C; 58.3 ± 2.8 vs. 48.4 ± 2.7; *P* < 0.001)], on the 2nd day [as shown by EL (40.9 ± 12.2 vs. 18.2 ± 8.4; *P* = 0.002) and DM (662.5 ± 139.3 vs. 317.8 ± 100.4; *P* = 0.001) and; MDP (53.7 ± 3.4 vs. 42.0 ± 3.3; *P* < 0.001)], on the 3rd day [as shown by MDP (44.5 ± 3.2 vs. 38.3 ± 3.4; *P* = 0.010)] and on the 4th day [as shown by EL (17.2 ± 7.0 vs. 8.0 ± 2.1; *P* = 0.006) and MDP (45.7 ± 3.6 vs. 35.4 ± 3.1; *P* < 0.001)]. Swim speed was not different between cold-exposed female and control female rats, except for that on the 3rd day (*P* = 0.038). Significant differences between control male and female rats were not observed in any learning parameter measured. Under cold stress gender difference was found in DM on the 4th day (*P* < 0.015) and SS on each day (*P*s < 0.003). Summed up, these data demonstrate that adult female rats are more sensitive to cold exposure in hippocampus-dependent spatial learning task than male rats.

Regarding spatial accuracy and the ability to remember the location of the hidden platform, the results showed that the time spent by the rat in the target quadrant (TS) was significantly above the 25% chance level for male controls (37.33 ± 8.89%; *t*_(11)_ = 4.800, *P* = 0.001; one-sample *T* test), female control rats (35.17 ± 11.03%; *t*_(11)_ = 3.194; *P* = 0.009), cold-exposed male rats (30.08 ± 6.11%; *t*_(9)_ = 2.628, *P* = 0.027) and cold-exposed female rats (31.11 ± 5.19%; *t*_(9)_ = 3.719, *P* = 0.005; Figure 2E).

The time spent in the target quadrant (Figure 2E) and mean distance to the platform point origin was previously located are used as a measurement of the retrieval performance of a rat (Figure 2F). Two factor ANOVA showed a significant Treatment Effect (*F*_(1,43)_ = 5.004; *p* = 0.031) with non significant Gender Effect (*F*_(1,43)_ = 0.049; *p* = 0.826) or Interaction Gender × Treatment (*F*_(1,43)_ = 0.396; *p* = 0.533). The LSD *post hoc* test showed that cold-exposed male rats spent less time in the target quadrant than control male rats (*P* = 0.049). A significant difference between cold-exposed and control female rats was not found (*P* > 0.05). Globally, cold exposure caused a minimal impairment in hippocampus-dependent spatial memory in male rats as shown by less time spent in the target quadrant. Mean distance to platform, the other retrieval parameter, however, was not affected by Treatment (*F*_(1,43)_ = 0.067; *p* = 0.797), Gender (*F*_(1,43)_ = 0.013; *p* = 0.909) or Interaction Treatment × Gender (*F*_(1,43)_ = 2.619; *p* = 0.114).

### Electrophysiology

**Figure 3:**
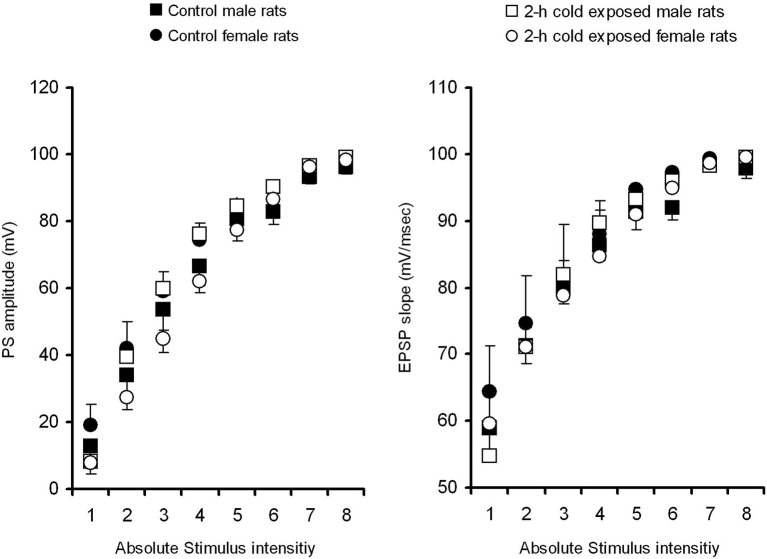
**Absolute input–output curves of the population spike (PS) amplitude (left) and field excitatory postsynaptic potential (EPSP) slope (right) in the dentate gyrus (DG) area of the control (male, black square, *n* = 6; female, black circle, *n* = 6) and intermittent cold stress (male, empty square, *n* = 6; female, empty circle, *n* = 6) groups as a function of stimulus intensity before induction of LTP**. Bars are standard errors of the means.

To determine whether behavioral changes in response to cold stress are parallel with neuronal activity, we investigated the baseline and stimulated activity of the DG. In order to verify if the exposure to intermittent cold stress influenced the basal circuitry properties of the DG, average EPSP slopes and PS amplitudes were plotted against stimulus intensities of 100–1500 μA (I/O curves, Figure 3). Repeated-measures ANOVAs with Treatment and Gender as between-subjects factors showed that neither cold exposure (*F*s_(7,140)_ = 1.638 and 1.049) nor gender (*F*_(7,140)_ = 0.122 and 1.015) had a significant effect on PS amplitude across the stimulus intensity range (*p* > 0.05). Moreover, Interaction effect Treatment × Gender (*F*s_(7,140)_ = 1.168 and 1.113) did not reach a significant level. These results show that cold stress does not change I/O curves, indicating no overall change in the baseline transmission of the Perforant Pathway—DG synapsis.

#### Cold exposure impairs PS-LTP in male rats

**Figure 4:**
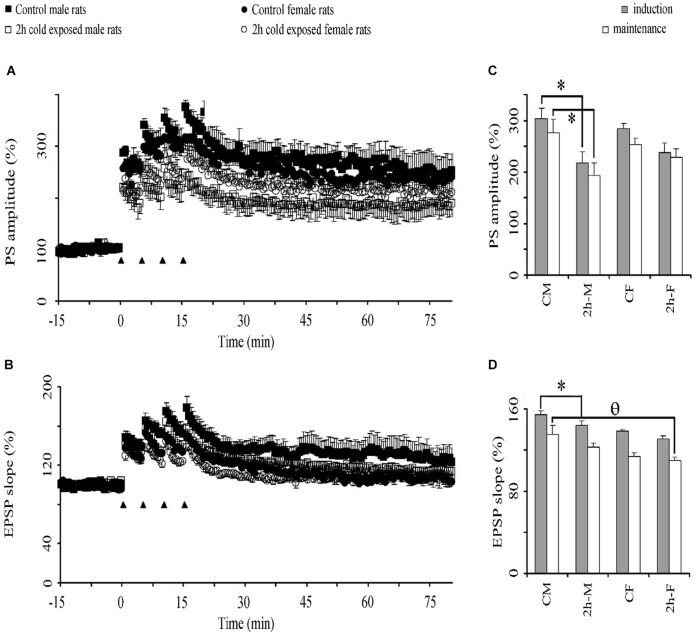
**The effect of cold exposure on time course of population spike (PSs) (A) and excitatory postsynaptic potentiation (EPSPs) (B) and graphical summaries of PS (C) and EPSP (D) potentiation in induction and maintenance phases**. A train of four consecutive 100 Hz tetanus stimuli (black triangles) induced potentiation of PS in the DG in all groups. Each symbol or bar represents the mean ± SE of six rats. * represents significant difference from PS amplitude of control male rats; θ significant difference from EPSP slope of control male rats.

High frequency stimulation-induced potentiation of basal PSs (Figure 4A) and EPSPs (Figure 4B) lasted at least 60 min in LTP recordings from all groups *in vivo*. This potentiation of PS amplitude was lower in the stress groups than in the control groups. Graphical summaries of PS and EPSP slope potentiation are shown in Figures 4C,D, respectively. Repeated-measures ANOVAs showed a significant Treatment Effect (*F*_(1,20)_ = 9.500; *P* = 0.006) and over intervals (*F*_2,40_= 181.151; *P* < 0.001) on PS amplitude, and a significant Gender Effect (*F*_1,20_ = 8.159; *P* = 0.010) on the fEPSP slope. LSD *post hoc* tests showed that 2-h cold exposure decreased PS potentiation during both induction (218.3 ± 21.6% vs. 304.5 ± 18.8%; *P* = 0.004) and maintenance intervals (193.9 ± 24.5% vs. 276.6 ± 25.4%; *P* = 0.015) in male rats, in contrast to the female rats. There was no difference in PS-LTP between both sexes; however, the fEPSP slope was more potentiated in control male rats in the induction interval (*P* = 0.026) and there was a trend (*P* = 0.061) in the maintenance interval. Under cold exposure, this gender dependency in the fEPSP slope was not observed (induction *P* = 0.072 and maintenance *P* = 0.262). We also observed, in the maintenance interval that cold exposure decreased EPSP slope potentiation in female rats when compared with male control rats (0.030). These results suggest that cold exposure has a negative effect on the synaptic plasticity of the DG neurons to the detriment of the male gender.

## Discussion

### Body weight and adrenal gland weight

Body weight loss or a reduction in body weight gain in growing rats may be an indication of disease, pain, stress or discomfort in an animal (Morton and Hau, 2002).

In the present study there were no significant differences in body weight gain between groups during any time period of the study. It explains that the environmental changes that the rats were exposed to had no impact on their body weight. Likewise, for the adrenal glands weight, there is no significant enlargement of these ones in stressed rats although a tendency is shown (0.07 ± 0.01 (mg organ/g of body weight); stressed group: 0.1 ± 0.02 (mg organ/g of body weight) *p* < 0.03). This may indicate that this kind of stress does not engage glucocorticoids as other stress paradigms.

Even though the noradrenergic system is postulated to play a primary role in an organism’s response to stress (Stanford, 1995). In fact in some studies it was well established that acute stress exposure can increase the discharge activity and norepinephrine (NE) release from noradrenergic locus coeruleus (LC) neurons (Korf et al., 1973; Abercrombie and Jacobs, 1987; Abercrombie et al., 1988). Furthermore, chronic exposure to stress can alter the response of LC neurons to subsequent stress exposure. However, to show the involvement of the Noradrenaline system in hippocampal pathway and also to explain its role in an organism’s response to stress we need to make a second study about Noradrenaline release in rats during prolonged cold-stress.

### Gender difference in behavioral performance in cold-exposed rats

The results of the present study suggest that intermittent cold exposure impairs hippocampal-dependent spatial memory in male rats but facilitates it in female rats. This is similar to the previous findings in restraint stress (Conrad et al., 2004). In the present study, cold-exposed female rats, significantly outperformed control female rats in the learning task to locate a hidden platform in a water maze on four consecutive days of testing; cold-exposed male rats, however, underperformed in the retrieval task on the probe trial. Female superiority in response to stress has been also reported in object recognition tasks (Beck and Luine, 2002) and in radial maze tests (Bowman et al., 2002).

To the best of our knowledge no study has yet reported the effects of cold stress on learning and memory in female rats. As the first study, we show that when a female rat is subjected to cold, spatial learning is improved. On the other hand there is a general agreement on impaired memory retrieval in males, rats (Panakhova et al., 1984; Nuñez et al., 2000) and humans (Coleshaw et al., 1983; O’Brien et al., 2007). Improved (Palinkas et al., 2005; Zheng et al., 2008) or unaffected (Baddeley et al., 1975) performance which was reported after cold exposure causing hypothermia is probably related to the variability in study designs, type (water or air), duration and intensity of cold exposure. Performance in spatial tasks seems to be correlated with the intensity of hypothermia. It was reported that spatial memory retrieval is resistant to mild hypothermia (30°C), but that it is severely impaired at body temperatures below 25°C (Panakhova et al., 1984). Since we found similar hypothermia after cold exposure in adult male and female rats, non significant decrease in time spent in the target quadrant observed in cold-exposed female rats suggests their resistance to hypothermia.

The results of the present study suggest a sex difference in motor ability to stress in rats as shown by faster swimming velocity in cold-exposed male rats. Since differences in SS create the possibility of a significant bias of ELs as measures of spatial learning, swim distance is the most appropriate measure of cognitive function in the MWM task (Liu et al., 2006). Therefore, increased swimming ability can explain the significant decrease on the first day and insignificant decreases on the remaining days in EL according to the control male rats in cold-exposed male rats.

Water temperature can be a confounding factor that might influence the acquisition rate of this task (Morris et al., 1982). For this reason we preferred not to let the rats swim in cold water. The present results are somewhat different from the experiment in which rats were exposed to cold during training trials. A quicker rate of acquisition observed in the rats trained at 19°C than in rats trained at 25°C water (Sandi et al., 1997) supports the present study; however better long-term retention is in contrast to the study.

Several studies demonstrated that exposure to 2°C ambient temperature did not decrease rectal temperature (Ahlers et al., 1991; Thomas et al., 1991). However, the results of the present study suggest that a longer lasting 4°C cold exposure procedure may be effective to produce hypothermia. Rectal temperature was measured as 32.7°C showing moderate hypothermia in the present study. Although not measured in the present study, it has been shown that exposure to ambient cold resulted in decreases in both memory and hippocampal temperature, suggesting that central cooling may be responsible for performance decrements (Ahlers et al., 1991). It may be suggested that a decrease in brain temperature slows hippocampal synaptic transmission, which would impair spatial memory (Moser and Andersen, 1994). Therefore hypothermia may be a possible explanation for impaired spatial memory performance after cold exposure in male rats. However, hypothermia could not be responsible for enhanced acquisition performance in female rats. We believe that neurohormonal response to stress should overcome the depressive effect of hypothermia on the hippocampus.

### Gender difference in behavioral performance in adult control rats

Numerous studies have suggested that sexual dimorphism may exist in learning and memory, particularly in types involving the hippocampus (Williams and Meck, 1991; Maren et al., 1994). Male rats outperformed female rats in tasks such as the radial-arm maze (Maren et al., 1994; Diamond et al., 1999) and the MWM (Brandeis et al., 1989; Maren et al., 1994), both of which require hippocampal spatial learning. However, no spatial superiority in male rats within Morris water task (MWT) was noted (Berger-Sweeney et al., 1995; Nicolle et al., 2003). Two studies have considered that female rats and mice are superior to males in learning some aspects of a working /reference memory version of the radial arm maze (Bimonte et al., 2000; Hyde et al., 2000). The present results suggest an equivalent performance in male and female rats tested at adult ages.

According to hormonal theory, higher testosterone levels lead to better spatial performance in males whereas varying estrogen levels result in variation in spatial learning and memory in females (Healy et al., 1999). In the present study we did not take into account the estrous cycle in female rats. One may presume that the changing number of female rats could be at any phase of the estrous cycle in all the trials of the MWM in our study; thus the effect of variations in gonadal hormone levels could be minimal in the MWM test. Moreover as the number of animal used is only nine, it is possible that the different phase of the estrous cycle could indeed influence the results.

### Gender difference in the dentate gyrus LTP in cold-exposed rats

Since hippocampus-dependent memory is mediated, at least in part, by hippocampal synaptic plasticity we also studied the electrophysiological properties of the Perforant Pathway—DG synapses. The examination of I/O functions across a range of stimulus strengths characterizes the efficiency of excitatory synaptic transmission. We found that cold exposure results in non-shifting I/O curves in adult anesthetized rats of both sexes indicating unaffected baseline transmission of Perforant Pathway—DG synapses. Although even short episodes of stress were reported to reduce the number of newly generated neurons in the DG (Schwabe and Wolf, 2013), acute cold stress (4 h, 4°C) was reported not to affect in synaptic efficacy in the DG of freely moving adult male rats (Bramham et al., 1998).

The PS-LTP induction was impaired in cold-exposed male rats, but not in female rats, in spite of similar I/O curves and similar drop in rectal temperature. Taken together with behavioral data, stress-induced memory disruption may therefore be related to suppression of LTP in male rats. Impaired LTP and poor retrieval performance in cold-exposed male rats are parallel. Therefore LTP in the DG seems to be intracellular correlates of retrieval performance in response to cold exposure. The association with LTP and the MWM is difficult, because the electrophysiological part of the study was carried out in urethane anesthetized rats. Indeed acute cold stress (1 time, 4°C and 90 min) leading to elevated serum corticosterone levels did not impair tetanus-evoked LTP in the DG of freely moving male rats (Bramham et al., 1998). The difference in the total exposure time of the two studies (90 min vs. 10 h) should be considered when interpreting these results. Although not measured in the present study, higher corticosterone levels would be expected in rats exposed to cold for a longer time. It is well-established that levels of corticosterone sufficient to occupy Type II glucocorticoid receptors produce a decrement in LTP, which is prevented by dehydroepiandrosterone (DHEA) in the DG of the hippocampus in male rats (Kaminska et al., 2000). Although the effects of cold exposure on histological changes in the DG were not investigated in the present studies, it was reported that chronically raised corticosterone reduced neurogenesis in the DG of the hippocampal formation (Karishma and Herbert, 2002; Wong and Herbert, 2006).

### Gender difference in the dentate gyrus LTP in control rats

A limited number of studies has examined sex difference in the LTP at the synapses of the rat hippocampus. A robust sex difference in the magnitude of LTP induced at the perforant path synapses was also reported in the CA1 region of hippocampal slices (Yang et al., 2004). As an earlier study these gender differences in LTP were specific to the EPSP component of the DG field potentials (Maren et al., 1994). Significant sex differences in EPSP amplitude were also observed, with slices from males having larger EPSP amplitudes than those from females (Zheng et al., 2008). However we did not find any difference either in baseline synaptic strength or in LTP between male and female adult rats in the DG. The results found here suggested that the LTP amplitude is not different depending on the gender.

## Conclusion

Overall, the results show that repeated cold exposure can selectively improve spatial learning in adult female rats, but impaired retention memory for platform location in male rats. It is possible that impaired LTP underlies some of the impaired retention memory caused by cold exposure in the male rats.

Cold exposure decreased the rectal temperature in both male and female rats. Nevertheless, there were no significant differences in body weight gain between groups during any time period of the study. It explains that the environmental changes that the rats were exposed to had no impact on their body weight.

We have followed the experimental protocol used in several studies on stress and the test of the levels of the principal sexual hormones was not explored here but could be assessed in further studies.

Data from the present study demonstrate that cold-exposed male rats show worse spatial memory performance and impaired PS-LTP, while cold-exposed female rats show enhanced learning ability and unaffected LTP. Detailed studies are needed to understand the gender dependent exact mechanism of the effects of cold stress on hippocampal pathways for learning and memory.

## Author and contributors

Hajar Elmarzouki has done the core experiments and the reports; Youssef Aboussaleh has submitted the project and planned the study; Cem Suer has supervised the work; Soner Bitiktas has assisted in animals models; Seda Artis has revised and did some data analysis; Nazan Dolu has followed the work; Ahmed Ahami has read the report and provided good advices.

## Conflict of interest statement

The Reviewer Dr. Mohamed Najimi declares that, despite having collaborated with the authors, the review process was handled objectively and no conflict of interest exists. The authors declare that the research was conducted in the absence of any commercial or financial relationships that could be construed as a potential conflict of interest.

## References

[B1] Abercrombie E. D. Jacobs B. L. (1987). Single-unit response of noradrenergic neurons in the locus coeruleus of freely moving cats. I. Acutely presented stressful and nonstressful stimuli. J. Neurosci. 7, 2837–2843 3625275 10.1523/JNEUROSCI.07-09-02837.1987 PMC6569145

[B2] Abercrombie E. D. Keller R. W. Jr. Zigmond M. J. (1988). Characterization of hippocampal norepinephrine release as measured by microdialysis perfusion: pharmacological and behavioral studies. Neuroscience 27, 897–904 10.1016/0306-4522(88)90192-3 3252176

[B3] Ahlers S. T. Thomas J. R. Berkey D. L. (1991). Hippocampal and body temperature changes in rats during delayed matching-to-sample performance in a cold environment. Physiol. Behav. 50, 1013–1018 10.1016/0031-9384(91)90430-v 1805262

[B4] Artis A. S. Bitiktas S. Taşkin E. Dolu N. Liman N. Suer C. (2012). Experimental hypothyroidism delays field excitatory post-synaptic potentials anddisrupts hippocampal long-term potentiation in the dentate gyrus of hippocampal formation and y-maze performance in adult rats: hypothyroidism delays fEPSP and disrupts spatial memory. J. Neuroendocrinol. 24, 422–433 10.1111/j.1365-2826.2011.02253.x 22070634

[B45] Baddeley A. D. Thomson N. Buchanan M. (1975). Word length and the structure of short-term memory. J. Verbal Learning Verbal Behav. 14, 575–589 10.1016/S0022-5371(75)80045-4

[B5] Beck K. D. Luine V. N. (2002). Sex differences in behavioral and neurochemical profiles after chronic stress: role of housing conditions. Physiol. Behav. 75, 661–673 10.1016/s0031-9384(02)00670-4 12020731

[B6] Berger-Sweeney J. Arnold A. Gabeau D. Mills J. (1995). Sex differences in learning and memory in mice: effects of sequence of testing and cholinergic blockade. Behav. Neurosci. 109, 859–873 10.1037/0735-7044.109.5.859 8554711

[B7] Bimonte H. A. Hyde L. A. Hoplight B. J. Denenberg V. H. (2000). In two species, females exhibit superior working memory and inferior reference memory on the water radial-arm maze. Physiol. Behav. 70, 311–317 10.1016/s0031-9384(00)00259-6 11006429

[B8] Bliss T. V. P. Collingridge G. L. (1993). A synaptic model of memory: long-term potentiation in the hippocampus. Nature 361, 31–39 10.1038/361031a0 8421494

[B9] Bowman R. E. Ferguson D. Luine V. N. (2002). Effects of chronic restraint stress and estradiol on open field activity, spatial memory and monoaminergic neurotransmitters in ovariectomized rats. Neuroscience 113, 401–410 10.1016/s0306-4522(02)00156-2 12127097

[B10] Bramham C. R. Southard T. Ahlers S. T. Sarvey J. M. (1998). Acute cold stress leading to elevated corticosterone neither enhances synaptic efficacy nor impairs LTP in the dentate gyrus of freely moving rats. Brain Res. 789, 245–255 10.1016/s0006-8993(97)01265-1 9573376

[B11] Brandeis R. Brandys Y. Yehuda S. (1989). The use of the Morris Water Maze in the study of memory and learning. Int. J. Neurosci. 48, 29–69 10.3109/00207458909002151 2684886

[B12] Britton K. T. Segal D. S. Kuczenski R. Hauger R. (1992). Dissociation between in vivo hippocampal norepinephrine response and behavioral/neuroendocrine responses to noise stress in rats. Brain Res. 574, 125–130 10.1016/0006-8993(92)90808-m 1638389

[B13] Coleshaw S. R. Van Someren R. N. Wolff A. H. Davis H. M. Keatinge W. R. (1983). Impaired memory registration and speed of reasoning caused by low body temperature. J. Appl. Physiol. Respir. Environ. Exerc. Physiol. 55(1 Pt. 1), 27–31 10.1097/00132586-198408000-00077 6885583

[B14] Conrad C. D. Jackson J. L. Wieczorek L. Baran S. E. Harman J. S. Wright R. L. (2004). Acute stress impairs spatial memory in male but not female rats: influence of estrous cycle. Pharmacol. Biochem. Behav. 78, 569–579 10.1016/j.pbb.2004.04.025 15251266

[B15] Diamond D. M. Park C. R. Heman K. L. Rose G. M. (1999). Exposing rats to a predator impairs spatial working memory in the radial arm water maze. Hippocampus 9, 542–552 10.1002/(sici)1098-1063(1999)9:5<542::aid-hipo8>3.0.co;2-n 10560925

[B47] Healy S. D. Braham S. R. Braithwaite V. A. (1999). Spatial working memory in rats: no differences between the sexes. Proc. Biol. Sci. 266, 2303–2308 10.1098/rspb.1999.0923 10629980 PMC1690445

[B46] Hyde L. A. Sherman G. F. Denenberg V. H. (2000). Non-spatial water radial-arm maze learning in mice. Brain Res. 863, 151–159 10.1016/s0006-8993(00)02113-2 10773203

[B16] Jonasson Z. (2005). Meta-analysis of sex differences in rodent models of learning and memory: a review of behavioral and biological data. Neurosci. Biobehav. Rev. 28, 811–825 10.1016/j.neubiorev.2004.10.006 15642623

[B17] Kaminska M. Harris J. Gijsbers K. Dubrovsky B. (2000). Dehydroepiandrosterone sulfate (DHEAS) counteracts decremental effects of corticosterone on dentate gyrus LTP. Implications for depression. Brain Res. Bull. 52, 229–234 10.1016/s0361-9230(00)00251-3 10822166

[B18] Karishma K. K. Herbert J. (2002). Dehydroepiandrosterone (DHEA) stimulates neurogenesis in the hippocampus of the rat, promotes survival of newly formed neurons and prevents corticosterone-induced suppression. Eur. J. Neurosci. 16, 445–453 10.1046/j.1460-9568.2002.02099.x 12193187

[B19] Korf J. Aghajanian G. K. Roth R. H. (1973). Increased turnover of norepinephrine in the rat cerebral cortex during stress: role of the locus coeruleus. Neuropharmacology 12, 933–938 10.1016/0028-3908(73)90024-5 4750561

[B20] Kvetnanský R. Pacák K. Sabban E. L. Kopin I. J. Goldstein D. S. (1997). Stressor specificity of peripheral catecholaminergic activation. Adv. Pharmacol. 42, 556–560 10.1016/s1054-3589(08)60811-x 9327962

[B21] Kvetnansky R. Sabban E. L. Palkovits M. (2009). Catecholaminergic systems in stress: structural and molecular genetic approaches. Physiol. Rev. 89, 535–606 10.1152/physrev.00042.2006 19342614

[B22] Liu C. Z. Yu J. C. Cheng H. Y. Jiang Z. G. Li T. Zhang X. Z. (2006). Spatial memory performance and hippocampal neuron number in osteoporotic SAMP6 mice. Exp. Neurol. 201, 452–460 10.1016/j.expneurol.2006.04.025 16839549

[B23] Maren S. De Oca B. Fanselow M. S. (1994). Sex differences in hippocampal long-term potentiation (LTP) and Pavlovian fear conditioning in rats: positive correlation between LTP and contextual learning. Brain Res. 661, 25–34 10.1016/0006-8993(94)91176-2 7834376

[B24] Morris R. G. Garrud P. Rawlins J. N. O’Keefe J. (1982). Place navigation impaired in rats with hippocampal lesions. Nature 297, 681–683 10.1038/297681a0 7088155

[B25] Morton D. B. Hau J. (2002). “Welfare assessment and humane endpoints,” in Handbook of Laboratory Animal Science (Vol. 1), eds Hau J. Van Hoosier L. Jr. (Boca Raton: CRC Press), 457–486

[B26] Moser E. I. Andersen P. (1994). Conserved spatial learning in cooled rats in spite of slowing of dentate field potentials. J. Neurosci. 14, 4458–4466 8027788 10.1523/JNEUROSCI.14-07-04458.1994 PMC6577035

[B27] Nicolle M. M. Prescott S. Bizon J. L. (2003). Emergence of a cue strategy preference on the water maze task in aged C57B6 x SJL F1 hybrid mice. Learn. Mem. 10, 520–524 10.1101/lm.64803 14657263 PMC305467

[B28] Nisenbaum L. K. Zigmond M. J. Sved A. F. Abercrombie E. D. (1991). Prior exposure to chronic stress results in enhanced synthesis and release of hippocampal norepinephrine in response to a novel stressor. J. Neurosci. 11, 1478–1484 1674004 10.1523/JNEUROSCI.11-05-01478.1991 PMC6575330

[B29] Nuñez J. L. Koss W. A. Juraska J. M. (2000). Hippocampal anatomy and water maze performance are affected by neonatal cryoanesthesia in rats of both sexes. Horm. Behav. 37, 169–178 10.1006/hbeh.2000.1572 10868480

[B30] O’Brien C. Mahoney C. Tharion W. J. Sils I. V. Castellani J. W. (2007). Dietary tyrosine benefits cognitive and psychomotor performance during body cooling. Physiol. Behav. 90, 301–307 10.1016/j.physbeh.2006.09.027 17078981

[B31] Palinkas L. A. Mäkinen T. M. Pääkkönen T. Rintamäki H. Leppäluoto J. Hassi J. (2005). Influence of seasonally adjusted exposure to cold and darkness on cognitive performance in circumpolar residents. Scand. J. Psychol. 46, 239–246 10.1111/j.1467-9450.2005.00453.x 15842414

[B32] Panakhova E. Burešová O. Bures J. (1984). The effect of hypothermia on the rat’s spatial memory in the water tank task. Behav. Neural Biol. 42, 191–196 10.1016/s0163-1047(84)91059-8 6525145

[B33] Rintamaki H. (2005). “Protective clothing and performance in cold environments”, in Proceeding of the Third International Conference on Human-Environment System (Tokyo, Japan).

[B34] Rosenzweig E. S. Barnes C. A. (2003). Impact of aging on hippocampal function: plasticity, network dynamics and cognition. Prog. Neurobiol. 69, 143–179 10.1016/s0301-0082(02)00126-0 12758108

[B35] Sandi C. Loscertales M. Guaza C. (1997). Experience-dependent facilitating effect of corticosterone on spatial memory formation in the water maze. Eur. J. Neurosci. 9, 637–642 10.1111/j.1460-9568.1997.tb01412.x 9153570

[B36] Sandi C. Woodson J. C. Haynes V. F. Park C. R. Touyarot K. Lopez-Fernandez M. A. (2005). Acute stress-induced impairment of spatial memory is associated with decreased expression of neural cell adhesion molecule in the hippocampus and prefrontal cortex. Biol. Psychiatry 57, 856–864 10.1016/j.biopsych.2004.12.034 15820706

[B48] Schwabe L. Wolf O. T. (2013). Stress and multiple memory systems: from ‘thinking’ to ‘doing’. Trends Cogn. Sci. 17, 60–68 10.1016/j.tics.2012.12.001 23290054

[B37] Stanford S. C. (1995). Central noradrenergic neurones and stress. Pharmacol. Ther. 68, 297–342 10.1016/0163-7258(95)02010-1 8719972

[B44] Suer C. Dolu N. Artis S. Aydogan S. (2009). Effects of carnosine on long-term plasticity of medial perforant pathway/dentate gyrus synapses in urethane-anesthetized rats: an in vivo model. Exp. Brain Res. 197, 135–142 10.1007/s00221-009-1899-x 19554317

[B38] Sutherland R. J. Whishaw I. Q. Regehr J. C. (1982). Cholinergic receptor blockade impairs spatial localization by use of distal cues in the rat. J. Comp. Physiol. Psychol. 96, 563–573 10.1037/h0077914 7119176

[B39] Thomas J. R. Ahlers S. T. Schrot J. (1991). Cold-induced impairment of delayed matching in rats. Behav. Neural Biol. 55, 19–30 10.1016/0163-1047(91)80124-w 1996945

[B40] Williams C. L. Meck W. H. (1991). The organizational effects of gonadal steroids on sexually dimorphic spatial ability. Psychoneuroendocrinology 16, 155–176 10.1016/0306-4530(91)90076-6 1961837

[B41] Wong E. Y. H. Herbert J. (2006). Raised circulating corticosterone inhibits neuronal differentiation of progenitor cells in the adult hippocampus. Neuroscience 137, 83–92 10.1016/j.neuroscience.2005.08.073 16289354 PMC2651634

[B42] Yang D. W. Pan B. Han T. Z. Xie W. (2004). Sexual dimorphism in the induction of LTP: critical role of tetanizing stimulation. Life Sci. 75, 119–127 10.1016/j.lfs.2003.12.004 15102526

[B43] Zheng G. Chen Y. Zhang X. Cai T. Liu M. Zhao F. (2008). Acute cold exposure and rewarming enhanced spatial memory and activated the MAPK cascades in the rat brain. Brain Res. 1239, 171–180 10.1016/j.brainres.2008.08.057 18789908

